# Integrated serum metabolomics and MRI biomarkers for MASLD: a practical framework for noninvasive staging and monitoring

**DOI:** 10.1007/s00261-026-05573-3

**Published:** 2026-05-26

**Authors:** Hamzah M. Alghzawi, Sweta Sahu, Aditya Rana, Rhobi Mwita, Sri Gundapaneni, Dushyant Dahiya

**Affiliations:** 1 Vanderbilt University Medical Center, Nashville, TN, United States; 2 School of Nursing, Tennessee State University, Nashville, TN, United States; 3 JJM Medical College, Davanagere, India; 4 Department of Internal Medicine, Army Hospital Research and Referral, New Delhi, India; 5 Sakarya University, Adapazari, Turkey; 6 UCF/HCA Florida Ocala, Ocala, United States; 7 School of Medicine, University of Kansas, Lawrence, United States

**Keywords:** MASLD, Metabolomics, MRI-PDFF, MR elastography, Noninvasive biomarkers

## Abstract

**Background:**

Metabolic dysfunction-associated steatotic liver disease (MASLD) affects nearly one in four adults globally, yet early diagnosis remains challenging due to the limitations of current diagnostic modalities. While liver biopsy is the reference standard, its invasiveness limits its utility for population screening. Similarly, single-modality noninvasive tests often fail to distinguish inflammation from fibrosis or detect early metabolic injury. This review synthesized recent evidence (2020–2025) regarding the integration of serum metabolomic profiling with quantitative magnetic resonance imaging (MRI) biomarkers to enhance diagnostic precision.

**Results:**

Emerging data indicate that serum metabolite panels, which detect upstream metabolic dysregulation, are highly complementary to MRI-PDFF (steatosis quantification) and MR Elastography (stiffness/fibrosis measurement). Integrated panels have demonstrated Area Under the Receiver Operating Characteristic (AUROC) values exceeding 0.90 for detecting advanced fibrosis, outperforming individual methods.

**Conclusions:**

We propose a three-tier clinical framework for integrating these biomarkers: transitioning from simplified biochemical screening in primary care to comprehensive multimodal staging in specialist centers, followed by risk-adapted longitudinal monitoring. This integrated approach offers a pathway toward precision hepatology, enabling earlier intervention and more accurate disease tracking without reliance on invasive procedures.

## Background

Metabolic dysfunction-associated steatotic liver disease (MASLD) encompasses a spectrum of hepatic disorders driven by systemic insulin resistance, obesity, and dyslipidemia. With a global prevalence approaching one in four adults, MASLD has emerged as a major public health concern due to its progressive nature and strong link to cardiometabolic complications [1–4]. Metabolomic studies reveal early biochemical derangements, such as altered fatty acids, bile acids, acylcarnitines, sphingolipids, and eicosanoids, that correlate with steatohepatitis and fibrosis progression [2, 4]. Up to 73% of individuals with metabolic steatosis harbor NAFLD [5], yet 70–90% remain undiagnosed because clinicians frequently rely on normal transaminase levels, despite nearly half of MASLD patients exhibiting normal enzyme levels [6–8]. Given that fibrosis stage is the principal determinant of outcomes, early detection and accurate noninvasive risk stratification are critical [9, 10]. Importantly, metabolomic biomarkers are subject to substantial biological and analytical variability arising from preanalytical factors, including fasting status and sample handling, inter-platform differences between LC-MS and NMR technologies, and inconsistent normalization pipelines [11–13]. Advanced MRI techniques such as MR elastography, while highly accurate, face significant accessibility barriers in resource-constrained settings due to specialized hardware requirements, prolonged acquisition times, and high cost [14]. A clear distinction must also be maintained between simple MASLD, metabolic dysfunction-associated steatohepatitis (MASH), and advanced fibrosis, as biomarker sensitivity and specificity vary substantially across these phenotypic stages [3, 11].

Although biopsy remains the reference standard, its invasiveness, sampling variability, and limited feasibility for population-level screening restrict its utility. Imaging modalities such as ultrasound elastography and MRI-proton density fat fraction (MRI-PDFF) quantify hepatic fat or stiffness but cannot distinguish inflammation from fibrosis or detect early biochemical injury [3, 15]. Serum indices such as FIB-4 and the MASLD Fibrosis Score demonstrate moderate discriminative performance, with AUC values of approximately 0.70–0.80, but are affected by inflammatory comorbidities [5]. Recent systematic reviews emphasize this diagnostic gap, noting that current approaches often miss the window for early intervention when lifestyle modifications are most effective [16, 17]. Conventional laboratory panels and ultrasonography lack sensitivity for early disease and cannot reliably grade fibrosis [18–22]. These limitations highlight the need for multimodal diagnostic paradigms that integrate biochemical and imaging information [23–25]. Biopsy itself carries a sampling error rate of up to 41%, inter-observer variability, and procedural risk [11, 26, 27].

Serum metabolomic profiling identifies upstream metabolic disruptions, while MRI provides quantitative anatomical and functional characterization of steatosis and fibrosis. Combining these complementary approaches may enable detection of both molecular dysfunction and tissue remodeling, enhancing diagnostic precision and longitudinal disease monitoring [2, 3, 15]. This review synthesized recent evidence from 2020 to 2025 on combined serum metabolite and/or proteomic panels and MRI biomarkers for MASLD staging and proposes a tiered, clinically feasible framework for their integration across diverse care settings. Literature was identified by systematic searches of PubMed, Scopus, and Web of Science from January 2020 to March 2025 using the terms “MASLD,” “metabolomics,” “MRI-PDFF,” “MR elastography,” and “noninvasive biomarkers.” Studies were included if they were peer-reviewed original research or systematic reviews reporting diagnostic or staging performance in human MASLD cohorts, with biopsy or established imaging reference standards. Studies with fewer than 20 participants were excluded. Priority was given to multicenter investigations and those reporting external validation cohorts.

## Current landscape

### Overview of MASLD pathophysiology

#### Metabolic dysregulation and hepatic steatosis

The pathogenesis of MASLD originates from systemic insulin resistance, which commonly develops in the setting of obesity and type 2 diabetes. Metabolomic profiling indicates that biochemical derangements emerge long before structural changes become apparent on imaging or histology. Early MASLD is characterized by elevated branched chain amino acids and altered phospholipid species, suggesting that metabolic dysregulation at the molecular level precedes morphologic liver injury [4]. This metabolic disturbance exerts a dual hepatic insult by stimulating de novo lipogenesis within hepatocytes while simultaneously impairing the suppression of lipolysis in adipose tissue. Impaired mitochondrial β oxidation and oxidative phosphorylation amplify this lipid overload, producing reactive oxygen species and activating cellular stress signaling cascades [28]. The resulting influx of free fatty acids to the liver leads to their esterification into triglyceride droplets that accumulate as hepatic steatosis [29].

#### Inflammation and immune activation

Persistent hepatocellular lipid accumulation provokes oxidative stress that releases damage-associated molecular patterns and proinflammatory cytokines, which in turn activate the innate immune system. Population-level evidence supports this mechanism. Analysis of NHANES data from 2017 to 2020 demonstrated that adults with MASLD exhibited progressive increases in serum C-reactive protein, interleukin-6, and tumor necrosis factor-α that paralleled both steatosis and fibrosis severity [30].

#### Fibrogenesis and clinical progression

The transition from inflammation to fibrosis represents a pivotal phase in MASLD pathogenesis. Identifying clinically significant fibrosis, defined as stage F2 or higher, is particularly important because it confers markedly increased risks of cirrhosis and hepatocellular carcinoma [3]. On the molecular level, serum PRO-C3, which reflects type III collagen formation, provides complementary insight into active fibrogenesis. The PRO-C3–based ADAPT algorithm achieves an AUROC of approximately 0.86 for advanced fibrosis, outperforming other serum scoring systems [8, 11, 31]. In a multicenter cohort of 447 adults with biopsy-proven disease, 35% progressed by at least one fibrosis stage over a median follow-up of 3.3 years. Among individuals with type 2 diabetes, the 4-year cumulative incidence of progression reached 24%, compared with 20% in those without diabetes [32]. Another paired biopsy study with approximately 21 months between assessments reported fibrosis progression in 26% of participants [33]. Magnetic resonance elastography demonstrated outstanding diagnostic performance, with meta-analytic AUROC values near 0.92 [34]. These data confirm the substantial risk of advancement and emphasize the need for structured, systematic monitoring strategies.

### Emerging and validation-stage biomarkers

The biomarker landscape in MASLD is expanding rapidly, with candidates at various stages of validation and clinical readiness. Among recent successes, thrombospondin-2 (TSP2) stands out as a validated marker. A multicenter European study in 2025 demonstrated that serum TSP2 outperformed established scores such as FIB-4, FAST, and Agile 3 for detecting advanced fibrosis in both clinic-based and community-based cohorts of patients with type 2 diabetes. Investigators combined TSP2 with PIIINP to create the TaP score, achieving robust discrimination for advanced fibrosis and illustrating a modern pathway from biomarker discovery to clinical implementation [35, 36]. Aptamer-based proteomic panels such as APTASHAPE showed early promise, with AUCs of 0.83 for MASH and 0.72 for distinguishing MASLD from controls in derivation cohorts, yet subsequent validation across independent and bariatric surgery populations produced variable results [37]. This divergence exemplifies reproducibility challenges that frequently impede biomarker translation.

Panels incorporating advanced glycation end products (AGEs) and their soluble receptor (esRAGE) alongside clinical variables demonstrated striking internal performance, achieving an AUC of 0.96 for MASLD identification in a 2025 study of 341 participants [38, 39]. However, broader multiethnic validation and standardized assay protocols remain essential. Additional molecular strategies, including eicosanoid lipidomics and immune gene expression panels, showed encouraging discrimination in early analyses [14, 40], though prospective multicenter validation is lacking. A notable 2025 innovation was the GP Index, an age-independent fibrosis staging tool combining circulating glutamine and propionate levels that outperformed FIB-4 and FIB-3 scores, particularly among older adults in whom traditional scores often yield false positives [41]. By targeting metabolites reflective of intrinsic hepatic function and gut–liver axis dynamics, the GP Index illustrates how focused biomarker design can overcome known clinical limitations. Multiomics approaches integrated through artificial intelligence frameworks have yielded further gains, with machine-learning models incorporating six proteins, including CNPY4, ENTPD6, and HLA-A, and eight clinical variables achieving AUCs of 0.94 in training cohorts and 0.88 in external validation for MASLD diagnosis [42].

### Serum metabolite biomarkers

Circulating metabolite profiles vary meaningfully across the MASLD spectrum, providing biochemical signatures that correlate with disease stage. In early steatosis, before histologic or imaging abnormalities appear, studies commonly identify modest elevations in branched chain amino acids together with shifts in phosphatidylcholine and lysophosphatidylcholine classes, patterns that strongly correlate with insulin resistance [4]. Advanced fibrosis brings a further shift toward markers of fibrogenesis and declining hepatocellular synthetic capacity, including increased PRO-C3 and ADAPT scores as well as disturbances in methionine and glutathione metabolism [4, 11, 43]. The transition to steatohepatitis is marked by greater metabolic perturbations, particularly enrichment of inflammatory and oxidative stress lipid mediators such as oxylipins and eicosanoids [40].

Despite these advances, metabolomic biomarkers remain limited by both analytical and biological variability. Preanalytical differences in sample collection, storage, and handling can influence metabolite stability, while discrepancies between liquid chromatography–mass spectrometry and nuclear magnetic resonance platforms introduce systematic variation. Inconsistent data processing pipelines across laboratories further compromise reproducibility [11–13]. Across published models, diagnostic performance varies widely, with reported AUCs typically ranging from 0.80 to above 0.90 in well-phenotyped research cohorts [40, 44]. Head-to-head comparisons between metabolomic panels and MRI-PDFF yield similarly broad results, with AUCs from 0.70 to 0.90 depending on endpoints and populations [45]. This variability reflects differences in reference standards, inclusion criteria, and analytic methods [11, 44, 45]. Best-practice guidelines therefore emphasize rigorous standardization of preprocessing, normalization, and cross-platform benchmarking to minimize batch effects and enhance external validity. Until such harmonized protocols become routine, translating metabolomic discoveries into clinical diagnostics will remain constrained.

### MRI-based biomarkers

Proton density fat fraction (PDFF) measured by chemical shift encoded MRI provides a precise quantitative assessment of hepatic steatosis by calculating the proportion of fat signal relative to total hepatic signal. PDFF correlates closely with histologic steatosis grade and can detect hepatic fat content as low as 5%, a sensitivity that outperforms routine imaging. Magnetic resonance elastography (MRE) quantifies liver stiffness as a direct surrogate of fibrosis severity, with meta-analyses and multicenter studies consistently showing AUCs exceeding 0.90 for detection of advanced fibrosis, defined as stage F3 or higher [3]. Additional quantitative MRI techniques such as T1 mapping and diffusion-weighted imaging (DWI) provide complementary information. T1 mapping correlates with hepatic inflammation and early fibrosis [15], whereas DWI examines tissue water dynamics and typically shows reduced apparent diffusion coefficients in fibrotic liver [14]. Multiparametric MRI protocols combining PDFF, MRE, T1 mapping, and MR spectroscopy offer the possibility of comprehensive, single-session evaluation of steatosis, inflammation, and fibrosis, though they require longer acquisition times and sophisticated postprocessing that may limit practical use [14].

Regarding acquisition protocols, MRI-PDFF is typically performed using a six-echo gradient-echo sequence at 1.5 T or 3 T with fat–water decomposition algorithms, such as IDEAL-IQ or mDIXON. MRE employs a pneumatic driver delivering 60-Hz mechanical waves, with two-dimensional gradient-echo or spin-echo EPI readouts and automated stiffness maps generated via inversion algorithms. Key imaging pitfalls include iron overload causing T2* signal loss that confounds PDFF estimation, body habitus limiting MRE wave penetration, and ascites reducing MRE reliability. Interobserver variability in region-of-interest placement can affect stiffness measurements by 5%–15%, underscoring the need for standardized acquisition and reading protocols [14]. Emerging radiomics and AI-driven image analysis tools are under active investigation as a means of reducing reader dependency and extracting additional prognostic features from routine MRI acquisitions [14, 15]. Technical reproducibility of PDFF is excellent, with coefficients of variation below 5%, making it particularly valuable for longitudinal monitoring of therapeutic response. Diagnostic accuracy for early steatosis can reach AUC values up to 0.97, although accuracy diminishes slightly at very high fat fractions, likely owing to signal saturation [39, 44, 45]. Compared with ultrasound-based elastography, MRE is less operator dependent and exhibits superior reproducibility across scanners. While sensitivity for early fibrosis, from F1 to F2, is somewhat lower, MRE remains the most accurate and validated noninvasive modality for identifying clinically significant fibrosis in MASLD [46].

### MRI-based monitoring: advantages and limitations

Magnetic resonance imaging provides several key advantages for assessing MASLD. It enables noninvasive, radiation-free quantification of hepatic steatosis and fibrosis with high reproducibility and sensitivity to longitudinal change [15]. Unlike needle biopsy, MRI evaluates the entire liver, thereby eliminating sampling error and capturing the heterogeneous distribution of steatosis and fibrosis typical of MASLD. Nevertheless, MRI approaches face important barriers to widespread use. The most significant is cost, as comprehensive MRI evaluations remain far more expensive than serum tests or ultrasound. Advanced sequences such as MRE and multiparametric MRI demand longer acquisition times, specialized hardware, and postprocessing expertise, which increase workflow complexity and reduce accessibility [14]. Contraindications such as metallic implants or severe claustrophobia may exclude certain patients, and persistent inter-platform variability across scanners underscores the urgent need for acquisition protocol harmonization to ensure consistent results [14].

### Comparative summary

The principal biomarker modalities available for MASLD evaluation differ in scope and limitations. MRI-PDFF offers precise, quantitative measurement of hepatic fat without radiation exposure but cannot evaluate inflammation or fibrosis and is less widely available [3, 15]. Serum metabolite panels detect early metabolic dysfunction with relatively low cost and broad accessibility, though they remain vulnerable to analytical variability and require standardized interpretation [4, 12, 13]. MRE provides quantitative stiffness assessment that correlates closely with fibrosis stage and yields whole-liver evaluation with low operator dependency, yet it is affected by confounding factors such as inflammation and ascites and demands costly, specialized equipment [8, 34]. Integrative serum plus MRI panels combine molecular and anatomical information, achieving AUROCs exceeding 0.90 for key staging distinctions and enabling early detection and precise disease monitoring [8, 22, 46–48]. However, they are resource intensive, involve complex data integration, and must overcome regulatory and interoperability challenges before broad clinical adoption becomes feasible [12, 47] (Table 1).

**Table 1:** Comparison of major biomarker modalities for the assessment of metabolic dysfunction associated steatotic liver disease (MASLD)

Biomarker modality	Advantages	Limitations
Serum metabolite panels	Detect early metabolic dysfunction [4]; relatively low cost [22]; sensitive to steatosis/inflammation [19, 39]	Platform variability; influenced by diet/comorbidities; requires standardization and expertise [42, 43]
MRI – PDFF	Quantitative, reproducible fat measurement; detects steatosis ≥ 5%; noninvasive/no radiation [3, 11]	Cannot assess inflammation or fibrosis [7]; higher cost and limited availability [7, 11, 24]
MRI – MRE	Quantitative stiffness correlating with fibrosis [7, 32]; low operator dependency [32]; whole-liver coverage	Affected by inflammation/ascites [32]; requires specialized hardware/software; costly [41]
Integrative serum + MRI panels	Combine molecular + anatomical data; AUROC > 0.90 for key stages; enable early detection & precise staging [38, 46, 48, 49]	High resource intensity; complex data integration; regulatory and interoperability hurdles [42, 47]

MASLD pathophysiology reflects a continuum from metabolic derangement to fibrosis [1, 7]. Individual biomarker classes, whether serologic or imaging, capture partial facets of this spectrum [8, 11]. Integrated serum plus MRI approaches hold the potential to deliver comprehensive, reproducible, and clinically actionable assessment once standardization and cost barriers are addressed [12, 46, 47, 49].

## Integrative panels: serum metabolite + MRI biomarkers

### Rationale for integration

#### Complementary strengths

The case for integrating serum metabolomic profiling with MRI-based assessments rests on their fundamentally complementary nature. MRI biomarkers offer direct anatomical and biomechanical assessment of the liver. Proton density fat fraction (PDFF) quantifies hepatic fat accumulation with high precision, while magnetic resonance elastography (MRE) measures tissue stiffness as a surrogate for fibrosis burden [3]. Metabolomic analyses detect systemic biochemical disturbances, including alterations in lipid species, branched chain amino acids, and bile acid profiles, that often appear before structural liver changes become evident. These circulating signatures reflect the upstream metabolic derangements that drive disease pathogenesis and provide early molecular indicators of hepatic stress [4, 40]. Integrating these molecular and imaging modalities enables a comprehensive evaluation of MASLD that spans the full continuum from metabolic dysfunction to structural remodeling.

#### Improved diagnostic accuracy and staging

A growing body of evidence supports the superiority of integrative approaches over individual modalities. A multicenter investigation published in Nature Communications found that an integrated proteomic and imaging panel achieved an AUROC of 0.92 for differentiating metabolic dysfunction-associated steatohepatitis (MASH) from simple steatosis, representing an 8% to 10% improvement over either method in isolation [49]. Machine-learning algorithms that incorporate both metabolite profiles and MRI variables have reported diagnostic accuracies exceeding 93% for identifying at-risk MASH [46]. The global multicenter analysis by Younossi and colleagues demonstrated that combining serum metabolomic risk scores with PDFF and MRE measurements improved the area under the receiver operating characteristic curve for advanced fibrosis detection from 0.90 with MRE alone to 0.94 [47].

### Evidence from clinical studies

Several investigations have evaluated integrated serum metabolite and MRI biomarker approaches for MASLD assessment. The table below summarizes key studies highlighting their diagnostic performance for advanced fibrosis and their application in longitudinal monitoring (Table 2).

**Table 2:** Key clinical studies evaluating integrated serum metabolite and MRI biomarkers for staging and monitoring MASLD

Study (Year)	Cohort & Design	Integrated Biomarkers	AUROC (Advanced Fibrosis)	Sensitivity/Specificity	Longitudinal Monitoring
Younossi et al., 2025 [48]	Global, 1,220 patients, cross-sectional with biopsy validation	Metabolomic score + PDFF + MRE	0.94	91%/88%	Not reported
Verschuren et al., 2024 [49]	Multicenter, 312 patients, dual cohorts	Proteomic panel (IGFBP7, SSc5D, Sema4D) + MRE	0.92	89%/85%	12-month follow-up showed parallel reductions in PDFF and biomarkers
Stefanakis et al., 2024 [46]	Multinational, 443 biopsy-proven patients, machine-learning model	Metabolomics + clinical parameters + PDFF	0.93	90%/87%	Six-month validation cohort showed stable performance
Bai et al., 2023 [38]	Single-center, 178 patients	Eicosanoid panel + PDFF	0.91	88%/82%	Not reported
Mukherjee and Im, 2025 [9]	Prospective, 410 patients, three-year follow-up	Amino acid and lipid panel + MRE	0.90	87%/84%	26% progressed by one fibrosis stage tracked by biomarkers and MRE

This table summarizes representative studies assessing combined molecular and imaging approaches for noninvasive MASLD evaluation. Across diverse study designs, integrative panels consistently achieve high diagnostic accuracy, with AUROC values of 0.90 or higher for advanced fibrosis, while emerging longitudinal data indicate their capacity to monitor disease progression and therapeutic response.

In a 3-year prospective study, Mukherjee and Im examined 410 patients using a targeted amino acid and lipid panel in combination with MRE. The integrated metrics maintained an AUROC of 0.90 with 87% sensitivity and 84% specificity throughout follow-up [9]. Fibrosis progression occurred in 26% of participants and was consistently captured by both serum and imaging markers, confirming the potential of these approaches for ongoing disease surveillance. Verschuren et al. [49] analyzed 312 patients divided into two validation cohorts using a proteomic biomarker panel comprising IGFBP7, SSc5D, and Sema4D in combination with MRE. The model achieved an AUROC of 0.92 with 89% sensitivity and 85% specificity, outperforming established scores including FIB-4, APRI, and transient elastography [49]. A subset of participants underwent repeat PDFF and biomarker assessment at 12 months, with changes in both correlating to histologic improvement or progression, suggesting utility for longitudinal follow-up.

Bai et al. [14] evaluated an eicosanoid lipid panel combined with PDFF in 178 patients and reported an AUROC of 0.91 for distinguishing MASH from simple steatosis with 88% sensitivity and 82% specificity [14]. Although longitudinal data were not available, the findings demonstrated excellent diagnostic discrimination. Stefanakis et al. [46] developed a machine-learning model in 443 biopsy-confirmed patients that integrated metabolomic, clinical, and PDFF data to identify at-risk MASH, defined as F2 to F4. The model achieved an AUROC of 0.93 with 90% sensitivity and 87% specificity and maintained robust performance in a 6-month validation cohort [46]. The study by Younossi et al. [47] enrolled 1,220 patients with MASLD from multiple international centers and evaluated a combined metabolomic risk score integrated with PDFF and MRE. For the detection of advanced fibrosis, defined as stage F3 or higher, the integrated panel achieved an AUROC of 0.94 with 91% sensitivity and 88% specificity, significantly outperforming MRE alone, which showed an AUROC of 0.90, sensitivity of 88%, and specificity of 82% [48]. It must be noted that these high AUROC values were generated predominantly in well-phenotyped, tertiary-center cohorts. Key methodological concerns include modest sample sizes in several studies, limited ethnic diversity, heterogeneous reference standards, and absence of external validation in most reports. Until multicenter prospective replication across diverse settings is available, these diagnostic performance figures should be regarded as promising but preliminary [11, 46]. Together, these investigations show that integrated serum metabolite and MRI biomarkers consistently achieve AUROCs of 0.90 or higher with high sensitivity and specificity for advanced fibrosis while demonstrating feasibility of tracking disease evolution and therapeutic response over time.

### Limitations and gaps

Despite promising findings, integrative serum–MRI studies remain limited by modest sample sizes and cohort heterogeneity. A major technical challenge is the lack of standardized protocols across the metabolomic analytic workflow. Sources of variability include preanalytical factors such as choice of serum versus plasma, fasting status, and sample processing time; differences between analytical platforms, including liquid chromatography–mass spectrometry versus nuclear magnetic resonance spectroscopy; and postanalytic data processing steps encompassing peak alignment, normalization, and batch correction. This methodological heterogeneity undermines reproducibility and currently limits establishment of universal diagnostic thresholds [11].

Reported cohorts include 1,220 patients in a global multicountry cohort [26], 463 biopsy-proven patients in a multicenter fibrosis progression analysis [32], 20 patients in the single-center lipidomic study by Bai et al. [14], 365 patients in a multicenter validation of noninvasive models [45], 443 patients in a multinational machine-learning development dataset [46], and 312 patients in the initial biomarker panel development study [49]. The machine-learning model by Stefanakis et al. [46], despite high performance, requires validation in independent populations beyond its 443-patient development dataset [46]. Variable representation of age, ethnicity, comorbidity burden, and fibrosis stages across these studies restricts generalizability to broader clinical populations.

Economic and logistical barriers further constrain clinical translation. High costs, infrastructure requirements, and need for expert interpretation limit advanced MRI techniques for MASLD assessment mainly to tertiary centers. Harmonized metabolomic workflows, rigorous quality-control programs, and certified reference materials, alongside strategies to improve MRI accessibility, remain prerequisites for reliable widespread clinical adoption.

## Translational framework for integrating serum and MRI biomarkers in MASLD management

### Conceptual model for integration in clinical workflow

The integration of serum metabolite and MRI biomarkers into the diagnostic and monitoring pathway for metabolic dysfunction-associated steatotic liver disease (MASLD) requires a structured, tiered framework that accommodates real-world variability in healthcare resources, technical expertise, and patient risk profiles. The overarching goal is to align diagnostic precision with accessibility, ensuring that high-fidelity tools are used judiciously without overwhelming clinical infrastructure.

We propose a three-tier model that progresses from simple biochemical screening in primary care to advanced multimodal evaluation in hepatology centers, followed by personalized longitudinal monitoring. Each tier escalates diagnostic complexity in proportion to disease risk. This stepwise approach reflects recommendations from recent population-based and mechanistic reviews emphasizing pragmatic, risk-adapted diagnostic pathways [16, 17]. Provisional decision thresholds guiding tier transitions include a composite serum metabolite risk score above the 75th population percentile triggering Tier 2 referral; MRE stiffness of 2.5 kPa or higher or MRI-PDFF of 10% or higher confirming significant hepatic involvement and entry into Tier 3 monitoring; and a stiffness increase of 0.5 kPa per year or greater shortening monitoring intervals to 6 months. In low- and middle-income country settings where MRE and advanced metabolomic platforms are unavailable, Tier 2 assessment may be approximated using FIB-4, MASLD Fibrosis Score, and point-of-care ultrasound elastography, with scalable digital metabolite panels representing a future cost-reduction strategy [11, 12, 14] (Table 3).

**Table 3:** Summarizes the framework structure and corresponding biomarker modalities, while Figure 1 (schematic representation) illustrates how patient flow transitions from population screening to specialty staging and ongoing surveillance

Tier	Target Population	Core Biomarkers/Methods	Primary Objective	Clinical Setting & Feasibility
Tier 1 Primary Care Screening	Adults with metabolic risk factors	Branched chain amino acids, phospholipid ratios, inflammatory markers [4]	Early identification and risk stratification	Routine lab tests; minimal infrastructure
Tier 2 Specialist Staging	High-risk or abnormal Tier 1 results	Expanded metabolomic panels + MRI-PDFF and MRE [48, 49]	Accurate steatosis/fibrosis staging	Specialty centers with MRI access
Tier 3 Longitudinal Monitoring	Diagnosed MASLD patients	Serial serum + MRI biomarkers [9]	Disease trajectory/treatment response monitoring	Risk-adapted interval (6–24 months)

Proposed Three Tier Framework for Integrating Serum Metabolite and MRI Biomarkers in MASLD Care

### Biomarker selection based on disease stage

#### Tier 1. Primary care screening

At the population level, individuals with obesity, type 2 diabetes, or dyslipidemia represent the principal at-risk cohort. Initial screening can employ simplified serum panels that quantify branched chain amino acids, selected phospholipid ratios, and inflammatory markers such as C-reactive protein and interleukin-6 [4].

#### Tier 2. Specialist staging

Patients with abnormal Tier 1 results or persistently elevated transaminases should undergo comprehensive staging that combines expanded metabolomic profiling with quantitative MRI techniques, specifically proton density fat fraction (PDFF) and magnetic resonance elastography (MRE). PDFF precisely quantifies hepatic steatosis, whereas MRE measures liver stiffness as a surrogate for fibrosis burden. In combination, these modalities have demonstrated AUROCs exceeding 0.90 for differentiating simple steatosis, MASH, and advanced fibrosis across multicenter and global cohorts [47, 49]. Specialist centers provide both the technical capacity and interpretive expertise required for these integrated assessments, establishing a robust foundation for individualized management planning.

#### Tier 3. Longitudinal monitoring

Once a diagnosis is established, the clinical focus shifts toward tracking disease trajectory and assessing therapeutic response. Serial measurement of serum and imaging biomarkers permits dynamic risk restratification. Evidence supports risk-adapted monitoring intervals approximately every 6 months for patients with advanced fibrosis or multiple metabolic comorbidities, and every 18–24 months for those with early-stage, metabolically stable disease [9]. This adaptive schedule optimizes resource use while maintaining appropriate surveillance intensity.

### Data fusion strategies for integrative assessment

The optimal method for combining biochemical, imaging, and clinical information remains an evolving domain. Two complementary strategies dominate current research:

1. *Algorithmic Integration* Machine-learning models, such as the categorical gradient boosting framework reported by Stefanakis et al. [46], can automatically synthesize high-dimensional metabolomic, imaging, and clinical variables into unified risk indices. These systems capture nonlinear relationships that conventional statistics may overlook, yielding superior predictive accuracy. Nevertheless, their deployment demands computational infrastructure, continuous model updating, and algorithmic transparency to satisfy clinical and regulatory standards.
2. *Clinical Scoring Integration* Simpler weighted scoring systems conceptually akin to the FIB-4 or NAFLD Fibrosis Score combine a limited number of serum and clinical parameters using explicit formulas [11]. Although such tools may achieve slightly lower accuracy than machine-learning models, they offer major advantages in interpretability, reproducibility, and cost effectiveness. Their transparency facilitates clinician acceptance and eases adoption across diverse healthcare environments. Together, these complementary strategies allow flexible implementation of the proposed framework across both resource-rich and resource-limited settings.

### Example use case: primary care → hepatology referral pathway

**Fig. 1:**
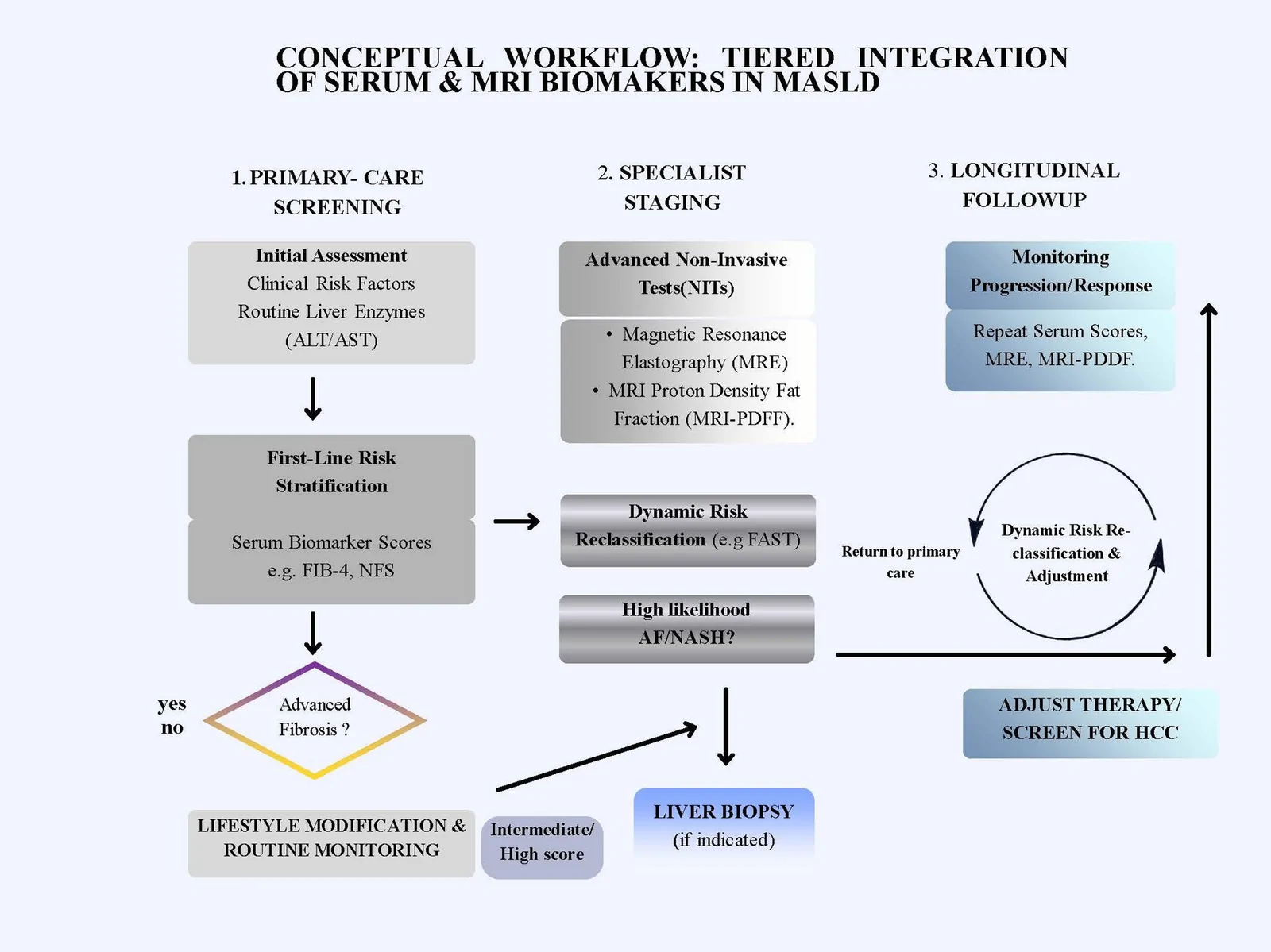
Conceptual workflow for tiered integration of serum and MRI biomarkers in MASLD. Schematic illustration showing the transition fromprimary care screening to specialist staging and longitudinal follow up, with feedback loops for dynamic risk reclassification

A practical example illustrates the translational workflow. A 45-year-old man with obesity, BMI 34 kg/m², type 2 diabetes, and mildly elevated ALT of 68 U/L undergoes Tier 1 screening with a simplified metabolite panel measuring branched chain amino acids and phospholipid ratios. The resulting composite risk score falls in the 75th percentile relative to reference populations, indicating probable MASLD. He is referred to a hepatology service for Tier 2 assessment, which includes expanded metabolomic profiling, PDFF, and MRE. Results reveal PDFF of 18%, indicating moderate steatosis; MRE of 3.2 kPa, indicating minimal fibrosis; and a metabolomic signature consistent with early steatohepatitis. The hepatologist initiates lifestyle intervention and emerging pharmacologic therapy, then enrolls the patient in Tier 3 longitudinal monitoring with annual serum plus MRI follow-up. If MRE stiffness or metabolomic inflammation indices rise on subsequent evaluation, the interval shortens to 6 months. This case exemplifies how the tiered framework ensures efficient escalation of diagnostic complexity, minimizes unnecessary imaging in low-risk individuals, and facilitates early identification of patients who benefit from intensified management (Fig. 1).

## Clinical implications and future directions

Integrated serum metabolite–MRI biomarker panels currently occupy a pivotal stage between research validation and real-world implementation. Cumulative evidence demonstrates diagnostic accuracy, with AUROCs ranging from 0.90 to 0.94, that approaches or exceeds the performance of liver biopsy for key endpoints, including fibrosis staging and MASH differentiation [47, 49]. Although these results are encouraging, translation into routine clinical practice requires several critical prerequisites, among which methodological standardization is central. Without this, the same patient could receive discordant risk scores depending on the testing site, undermining clinical trust and regulatory acceptance [11]. Furthermore, formal cost-effectiveness analyses remain scarce. Together, these considerations define the immediate agenda for enabling clinical readiness. In primary care, simplified metabolite panels have substantial potential for scalable population-level screening. Among individuals with metabolic comorbidities, blood-based metabolomic profiling could identify those at greatest risk for progressive liver disease [4]. This offers an efficient alternative to imaging-based screening, which remains impractical for large populations because of cost and access constraints.

Recent studies demonstrate how AI-driven integration can extend the power of combined biomarker datasets. A 2025 investigation applying Mendelian randomization across 2,925 proteomic markers identified six molecular biomarkers, including CNPY4, ENTPD6, and HLA-A, and eight clinical variables that, when integrated via random forest modeling, achieved an AUC of 0.941 in training and 0.875 in external validation for MASLD diagnosis [50]. Regulatory agencies increasingly recognize noninvasive composite biomarkers as acceptable surrogate endpoints for MASLD drug development. Integrated panels can quantify both structural changes, including fibrosis regression and steatosis reduction, and functional improvements, including metabolic normalization and inflammatory attenuation, within a single assay. These multimodal endpoints could shorten trial duration, reduce participant burden by avoiding repeat biopsies, and enhance sensitivity to early treatment effects, while maintaining alignment with clinically meaningful outcomes [5].

Large-scale, multicenter validation is imperative to confirm the generalizability of integrated biomarker panels. Future studies should emphasize demographic diversity, including racial and ethnic representation, varied metabolic phenotypes, and age strata, as well as standardized phenotyping across sites. Longer follow-up periods are critical for evaluating biomarkers and assessing outcomes such as cirrhosis, hepatocellular carcinoma, liver-related mortality, and cardiovascular events [46]. Advanced computational frameworks, especially deep neural networks, hold promise for extracting subtle, clinically relevant patterns from high-dimensional metabolomic and imaging data [10]. However, these systems must overcome challenges of data volume, external validation, and interpretability. Models require large, annotated training datasets to prevent overfitting, as well as explainable outputs that complement rather than replace clinical reasoning. Ongoing interdisciplinary collaboration among hepatologists, data scientists, and biostatisticians will be essential for translating algorithmic discoveries into actionable clinical tools.

**Fig. 2:**
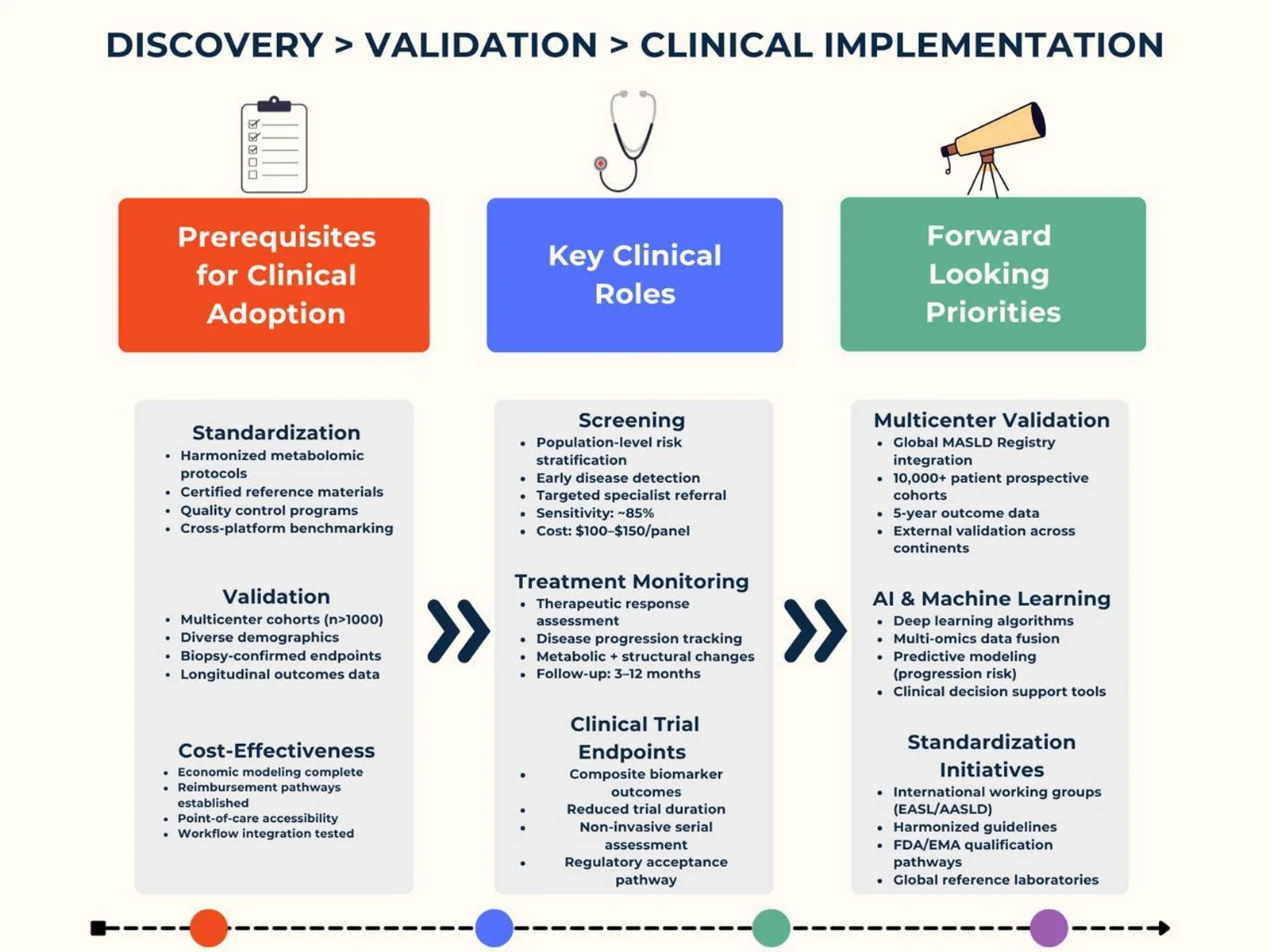
Clinical implications and future directions for integrated biomarker panels in MASLD. Infographic summarizing readiness for clinical adoption, key applications, and future research priorities. The left panel highlights prerequisites for implementation (standardization, validation, and cost effectiveness). The middle panel illustrates clinical roles in screening, treatment monitoring, and trial endpoints. The right panel outlines forward looking directions, including multicenter validation, AI integration, and global standardization initiatives representing the translational pathway from discovery to clinical practice

Professional societies and regulatory agencies should spearhead a unified standardization effort encompassing preanalytical, analytical, and postanalytical domains. Preanalytical standardization should harmonize blood collection and storage protocols, including fasting state, tube type, centrifugation, and freeze–thaw cycles. Analytical standardization should establish certified reference materials, quality-control metrics, and interlaboratory proficiency programs. Postanalytical standardization should define standardized interpretive thresholds and decision points to ensure consistent clinical application [11]. International consensus guidelines analogous to those for HbA1c or lipid testing would enhance reproducibility and foster widespread adoption. Without such coordination, the promise of metabolomic biomarkers may remain confined to academic research rather than achieving clinical translation (Fig. 2).

## Conclusions

Metabolic dysfunction-associated steatotic liver disease (MASLD) represents a dynamic intersection between systemic metabolic dysregulation and hepatic injury. The limitations of conventional diagnostic modalities including biopsy invasiveness, serum index variability, and imaging cost have driven the search for integrative approaches that unite molecular and structural perspectives. Evidence synthesized in this review demonstrates that combined serum metabolite and MRI biomarker**s** can deliver diagnostic precision comparable to histologic assessment while providing a reproducible, noninvasive, and patient-centered alternative. By capturing both metabolic activity and morphologic change, these integrated panels enable early detection, accurate staging, and longitudinal monitoring within a single framework. The emerging role of machine learning integration signals a vital shift, where multidimensional biomarker data can be included in clinically interpretable tools. Combined serum metabolite and MRI biomarker panels represent a validated, noninvasive, and patient-centered approach to MASLD diagnosis and monitoring. Realizing their full potential requires global standardization, multicenter validation, and collaborative regulatory alignment, paving the way for precision hepatology in routine practice.

## Data Availability

No datasets were generated or analysed during the current study.

## Abbreviations

ADAPT: Algorithm incorporating PRO-C3
AGEs: Advanced glycation end products
AI: Artificial intelligence
ALT: Alanine aminotransferase
APRI: Aspartate aminotransferase-to-platelet ratio index
AUC: Area under the curve
AUROC: Area under the receiver operating characteristic curve
BCAA: Branched-chain amino acid
CRP: C-reactive protein
DWI: Diffusion-weighted imaging
esRAGE: Endogenous secretory receptor for advanced glycation end products
FAST: FibroScan-AST score
FIB-4: Fibrosis-4 index
HbA1c: Hemoglobin A1c
IL-6: Interleukin-6
LC-MS: Liquid chromatography–mass spectrometry
MASLD: Metabolic dysfunction-associated steatotic liver disease
MASH: Metabolic dysfunction-associated steatohepatitis
MRE: Magnetic resonance elastography
MRI: Magnetic resonance imaging
MRI-PDFF: Magnetic resonance imaging-proton density fat fraction
NAFLD: Nonalcoholic fatty liver disease
NIT: Noninvasive test
NMR: Nuclear magnetic resonance
PDFF: Proton density fat fraction
PIIINP: Procollagen III N-terminal peptide
PRO-C3: Type III collagen formation marker
T2D: Type 2 diabetes
TNF-α: Tumor necrosis factor-alpha
TSP2: Thrombospondin-2
